# Delay in treatment intensification increases the risks of cardiovascular events in patients with type 2 diabetes

**DOI:** 10.1186/s12933-015-0260-x

**Published:** 2015-08-07

**Authors:** Sanjoy K Paul, Kerenaftali Klein, Brian L Thorsted, Michael L Wolden, Kamlesh Khunti

**Affiliations:** Clinical Trials and Biostatistics Unit, QIMR Berghofer Medical Research Institute, 300 Herston Road, Herston, Brisbane, QLD 4006 Australia; Novo Nordisk A/S, Vandtårnsvej, Denmark; Leicester Diabetes Centre, University of Leicester, Leicester, UK

**Keywords:** Type 2 diabetes, Delay in treatment intensification, Cardiovascular risk, Longitudinal analysis, Clinical inertia

## Abstract

**Background:**

The aim of the study was to evaluate the effect of delay in treatment intensification (IT; clinical inertia) in conjunction with glycaemic burden on the risk of macrovascular events (CVE) in type 2 diabetes (T2DM) patients.

**Methods:**

A retrospective cohort study was carried out using United Kingdom Clinical Practice Research Datalink, including T2DM patients diagnosed from 1990 with follow-up data available until 2012.

**Results:**

In the cohort of 105,477 patients mean HbA1c was 8.1% (65 mmol/mol) at diagnosis, 11% had a history of cardiovascular disease, and 7.1% experienced at least one CVE during 5.3 years of median follow-up. In patients with HbA1c consistently above 7/7.5% (53/58 mmol/mol, n = 23,101/11,281) during 2 years post diagnosis, 26/22% never received any IT. Compared to patients with HbA1c <7% (<53 mmol/mol), in patients with HbA1c ≥7% (≥53 mmol/mol), a 1 year delay in receiving IT was associated with significantly increased risk of MI, stroke, HF and composite CVE by 67% (HR CI: 1.39, 2.01), 51% (HR CI: 1.25, 1.83), 64% (HR CI: 1.40, 1.91) and 62% (HR CI: 1.46, 1.80) respectively. One year delay in IT in interaction with HbA1c above 7.5% (58 mmol/mol) was also associated with similar increased risk of CVE.

**Conclusions:**

Among patients with newly diagnosed T2DM, 22% remained under poor glycaemic control over 2 years, and 26% never received IT. Delay in IT by 1 year in conjunction with poor glycaemic control significantly increased the risk of MI, HF, stroke and composite CVE.

## Background

Currently 8.3% of adults worldwide are estimated to have diabetes [1]. The risk of cardiovascular complications has been related to glycaemia in patients with type 2 diabetes mellitus (T2DM). Randomised controlled trials have conclusively demonstrated that the risk of microvascular complications can be reduced by intensive glycaemic control in patients with T2DM [2–4]. However, there are controversies regarding the benefits of intensive glucose control [HbA1c <7% (53 mmol/mol)] on macrovascular events (CVE), as some of the large trials have failed to show any significant reduction in CVE [4, 5]. The ACCORD trial failed to show any benefit of intensive glucose lowering on CVE, although the haemoglobin A1c (HbA1c) level was reduced to 6.4% (46.4 mmol/mol) in the intensive treatment arm compared to HbA1c level of 7.5% (58.5 mmol/mol) in the standard treatment arm [6]. The primary care based randomised trial ADDITION reported only a small, non-significant reduction in the incidence of CVE and death associated with early intensive management of the disease [7]. However, the UKPDS Post Trial Monitoring Study demonstrated that intensive glucose control starting at the time of diagnosis of diabetes could be associated with a significantly decreased risk of myocardial infarction (MI) and death from any cause [8]. Also, the meta-analysis of four large cardiovascular outcome trials in patients with T2DM revealed that tighter glycaemic control was associated with 9% reduction in risk of major cardiovascular events [9]. However, tight glycaemic control was not associated with reduced mortality.

Glycaemic management in patients with T2DM has become increasingly complex, and in some cases controversial, with a widening classes of pharmacological agents now available [10–12]. Based on the individual characteristics of the patients, step-wise life-style and pharmacological approaches have been suggested by international guidelines for better glycaemic management in patients with T2DM [13–15].

The American Diabetes Association guidelines recommend starting metformin alongside lifestyle modifications at diagnosis, aiming for a HbA1c target of <7% (<53 mmol/mol) [15]. Additional oral anti-diabetes drugs (OADs) may be added if the HbA1c continues to remain above the recommended target of 6.5% (48 mmol/mol), and if HbA1c reaches ≥7.5% (≥58 mmol/mol), further intensification including the use of insulin is recommended [13, 16]. The intensification of anti-diabetes therapies also depends on individual patient’s characteristics including age, co-morbidities, the risk of hypoglycaemia, and provider and patient’s preferences [13]. However, a high proportion of people with T2DM fail to reach the recommended glycaemic targets for a considerable period of time post diagnosis of diabetes (glycaemic burden) [17–21]. Among those with poor glycaemic control [HbA1c ≥7% (≥53 mmol/mol)], an overwhelmingly large proportion of people do not receive intensified treatment in time. This “delay in treatment intensification”, also termed as *clinical inertia*, has been discussed by some studies [17–21]. A recent study based on 80,000 patients with T2DM from the United Kingdom primary care system reported that the average time to intensification to two oral anti-diabetes drugs (OADs) from one OAD among patients with HbA1c above 7% (53 mmol/mol) was about 3 years [21]. The aspects of glycaemic variability and treatment quality indicators and their association with macrovascular risk were evaluated by Penno et al. [22] and Sidorenkov et al. [23], respectively. Asche et al. [24] evaluated the clinical and economic benefits of early intensification of treatment with insulin in patients with T2DM, and reported significant benefits in terms of glycaemic management.

While studies have reported the real-world scenario in terms of intensification of treatments for hyperglycaemia among patients with poorly controlled glycaemia, the possible effect of delay in treatment intensification in conjunction with the dynamic changes in glycaemic control on the vascular risk factors, has not yet been studied to the best of our knowledge. The aims of this retrospective cohort study were to (1) explore the glycaemic control over 2 years post diagnosis of diabetes in relation to treatment intensification, and (2) evaluate the effect of the delay in treatment intensification in conjunction with guideline recommended glycaemic control on the of risk of MI, heart failure (HF), stroke, and composite of these three CVE.

## Methods

### Data source

The data for this retrospective cohort study was extracted from the United Kingdom Clinical Practice Research Datalink (CPRD), which is representative of the United Kingdom general population [25–27]. All information collected in the CPRD has been subjected to validation studies and been proven to contain consistent and high-quality data [26, 28].

Data were extracted from CPRD with a first identifiable record of diagnosis code for T2DM covering period from January 1990 with follow-up data to December 2012, with maximum possible follow-up time of 23 years. The confirmation for the incident diagnosis of T2DM was based on Read/Oxford Medical Information System Codes [29], supported by rigorous classification techniques [30, 31].

The following information was extracted: age, gender, smoking status (defined as current, ex or never smoker), body mass index (BMI), HbA1c, history of cardiovascular and renal diseases before the diagnosis of diabetes, and clinical events during follow-up, including cardiovascular diseases (MI, HF, stroke and coronary heart diseases), atherosclerosis, diabetic neuropathy, and renal complications, along with dates of events. Detailed information on OADs and insulin along with anti-hypertensive and cardio-protective medications (including use of ACE inhibitor, Beta Blocker, Statin and other concomitant medications) were obtained from prescriptions along with dates. The study cohort (n = 105,477) was based under the conditions: (1) read code for T2DM and at least two prescriptions for any OAD or insulin (as recorded in the primary care system) within 6 months of date of diagnosis of T2DM (prescription provided and recorded by general practice), (2) age ≥18 years at index date (date of diagnosis of T2DM), (3) complete information on age, sex, smoking status at index date, (4) a measure of HbA1c available within a 3-month window of the index date (HbA1c measured within 3 months before the date of first diagnosis code recorded), (5) minimum 2 years of follow-up before the occurrence of any CVE post diagnosis of diabetes, (6) availability of dates of prescriptions longitudinally for anti-diabetes drugs, (7) completeness of dates for all CVEs during post diagnosis follow-up. Choice of this cohort of patients with new diagnosis of T2DM ensures censoring for CVE in the first 2 years post diagnosis of diabetes, and 2 years window for treatment intensification with the availability of data on glycaemic control.

Clinical comorbidities prior to diagnosis of diabetes, including cardiovascular and renal diseases, were coded as present if they were diagnosed at any point between entry into CPRD and the index date. The clinical and laboratory measures were arranged longitudinally on the basis of 6-monthly windows. The 6-month windows were defined progressive from the index date, with 180 days immediately post index date defining first follow-up window.

The study was approved by the Independent Scientific Advisory Committee (Protocol no 13_062R2).

### Statistical methods

Treatment intensification (IT) was defined in two ways: (1) adding a second OAD (OAD2) or (2) adding insulin to the first OAD (OAD + INS). Time to IT was calculated by subtracting the index date from the first date of IT. The subjects who did not belong to OAD2 or OAD + INS groups were defined as “never intensified” category.

Missing HbA1c data (about 9% missing over 24 months post diagnosis) at 6-monthly window were imputed using multiple imputation technique (Bayesian MCMC approach) [32, 33]. The consistency of the imputed data with the original HbA1c data was verified. Adequate checks were in place to ensure that patients were not lost to follow-up for imputing missing values during 2 years post diagnosis. Patients were categorized by HbA1c below or above 7% (53 mmol/mol) and 7.5% (58 mmol/mol) consistently over 1 and 2 years post diagnosis to identify poor glycaemic control.

The composite CVE was based on the occurrence of either of MI, HF or stroke. To evaluate the effect of delay in IT on events, only those vascular events were considered which occurred after the time of first intensification (OAD2 or OAD + INS), apart from the condition of no occurrence of CVE during first 2 years post diagnosis of diabetes (to ensure minimum exposure time of 2 years). In this context, the “time to” individual events were calculated by subtracting the first event date (as appropriate) from the date when intensified treatment started. The analysis set for cardiovascular risk analysis included only those belonging to OAD2 or OAD + INS categories.

As time to treatment intensification (TTIT) is highly likely to be interacted with the glycaemic control over time, the interaction of TTIT with HbA1c categories over time was evaluated in terms of cardiovascular risks. To evaluate the interaction effect we constructed the following four groups: reference group—TTIT <12 months and HbA1c <7% (<53 mmol/mol) (consistently <7% over 12 months post diagnosis); group 1—TTIT ≥12 months and HbA1c ≥7% (≥53 mmol/mol); group 2—TTIT <12 months and HbA1c ≥7% (≥53 mmol/mol); group 3—TTIT ≥12 months and HbA1c <7% (<53 mmol/mol). The Group 1 reflects both clinical inertia and glycaemic burden together, while Group 2 reflects only the glycaemic burden. Multivariate Cox regression models were used to evaluate the effect of delay in IT in conjunction with poor glycaemic control consistently over 1 year post diagnosis, adjusting for age and HbA1c at diagnosis of diabetes, sex, smoking status, use of cardio-protective medications (Statin, ACE/ARB and Beta Blocker), any renal disease during follow-up, and the history of CVD before diagnosis of diabetes. The proportionality assumption in the models was tested, and stratified models were fitted with the quartiles of age at diagnosis of diabetes as the stratifying factor. Separate analyses were also conducted for patients with and without the history of CVE. Additional multivariate Cox regression models were also fitted with incomplete information on BMI, systolic blood pressure, LDL-cholesterol and total cholesterol at diagnosis (about 62,000 patients).

To evaluate the possible lack of treatment intensification in older patients (age >70 years at diagnosis of diabetes) and in patients with history of CVD and renal diseases, logistic regression models were fitted. The odds ratios (OR) and their 95% confidence intervals were presented. The likelihood of receiving intensified treatment in poorly controlled patients by categories based on time windows of diagnosis was also evaluated.

## Results

In the cohort of 105,477 patients: 56% were male, 62% current or ex-smokers, mean (SD) age at diagnosis of 61 (13) years, and 11% (n = 11,955) had history of CVD before the diagnosis of diabetes (Table 1). The distribution of HbA1c was highly skewed at diagnosis of diabetes, with mean (SD) and median (IQR) levels of 8.1 (2.2)% (65 mmol/mol) and 7.4 (6.5, 9.3)% [57 (48, 78) mmol/mol], respectively, and 62% patients had HbA1c ≥7% (≥53 mmol/mol) at diagnosis.

**Table 1:** Descriptive statistics on study parameters at diagnosis of diabetes and at follow-up

	Without previous CVD	With previous CVD	All patients
n	93,522	11,955	105,477
Male^a^	50,963 (55)	7,609 (64)	58,572 (56)
Age at diagnosis (years)^b^	60 (13)	68 (11)	61 (13)
Patients by year of diagnosis^a^			
1990–December 1999	1,893 (2)	201 (2)	2,094 (2)
January 2000–December 2004	28,551 (31)	3,741 (31)	32,292 (31)
January 2005–December 2009	52,317 (56)	6,647 (56)	58,964 (56)
January 2010	10,761 (12)	1,366 (11)	12,127 (12)
Smoking status^a^			
Current smoking	17,545 (19)	2,022 (17)	19,567 (19)
Ex-smoking	39,016 (42)	6,922 (58)	45,938 (44)
BMI at diagnosis (kg/m^2^)^b^	32 (7)	31 (6)	32 (7)
HbA1c at diagnosis (%)^b^	8.2 (2.2)	7.8 (2.0)	8.1 (2.2)
HbA1c at diagnosis (mmol/mol)^b^	66	62	65
HbA1C ≥7% (≥53 mmol/mol) post diagnosis^a^			
Consistently during 1 year post diagnosis	27,375 (29)	3,096 (26)	30,471 (29)
Consistently during 2 years post diagnosis	20,856 (22)	2,245 (19)	23,101 (22)
CVE during follow-up^a^			
MI	1,876 (2.01)	569 (4.76)	2,445 (2.32)
HF	2,295 (2.45)	898 (7.51)	3,193 (3.03)
Stroke	1,782 (1.91)	485 (4.06)	2,267 (2.15)
Any CVE	4,293 (6.64)	1,833 (8.39)	6,126 (7.08)
Renal disease^a^			
Before diagnosis	16,630 (17.78)	3,116 (26.06)	19,746 (18.72)
Post diagnosis	25,662 (27.44)	4,601 (38.49)	30,263 (28.69)
Duration of follow-up (years)^c^	5.0 (3.4, 7.2)	5.4 (3.5, 7.7)	5.3 (3.5, 7.7)
Medication^a^			
2 OADs	41,696 (45)	4,515 (38)	46,211 (44)
Insulin	8,778 (9)	1,004 (8)	9,782 (9)
OAD + insulin	8,380 (9)	954 (8)	9,334 (9)
Intensified treatment	44,042 (45)	4,012 (56)	48,036 (46)

^a^N (%).

^b^Mean (SD).

^c^Median (IQR).

In the study cohort 48,036 patients (46%) received intensified treatment (IT) during follow-up. Among those who received IT, the proportions with time to IT (TTIT) <6 months, <1 year, and <2 years were 26, 36, and 53%, respectively. The overall median/mean time (months) to receiving IT, at least 2 OADs (2OADs) and at least 3 anti-diabetes drugs (3ADDs) were 21/29, 22/29, and 43/48, respectively.

### The clinical inertia and glycaemic burden

**Fig. 1:**
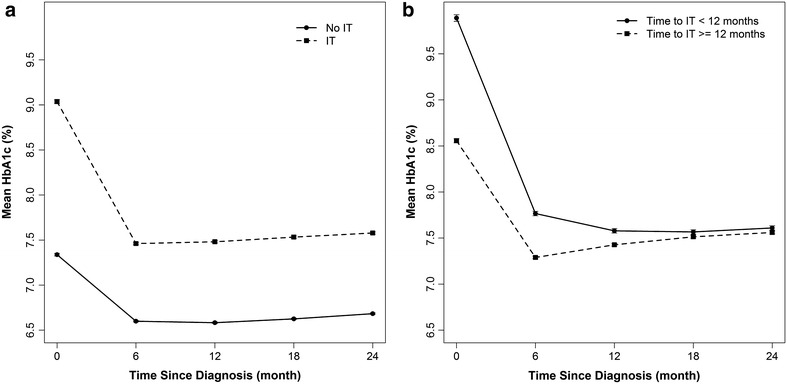
Six-monthly measure of HbA1c (mean and 95% CI) from diagnosis to 2 years, by **a** patients with and without intensified treatment during follow-up, **b** by patients receiving treatment intensification before or after 12 months of diagnosis.

The 6-monthly trajectory of HbA1c over 2 years post diagnosis of diabetes, by the categories of TTIT, is presented in Fig. 1. Patients who did not receive any IT during follow-up had their average glycaemic level below 7% (53 mmol/mol) during 2 years post diagnosis, starting with an average HbA1c level of 7.4% (57 mmol/mol) at diagnosis (Fig. 1a). However, patients who receive intensified treatment continued to have average HbA1c trajectory level around 7.5% (58 mmol/mol) during 2 years post diagnosis irrespective of the time of treatment intensification (Fig. 1a). Among patients with HbA1c ≥7% (≥53 mmol/mol) consistently during 1/2 year post diagnosis, 29/26% never received any IT during follow-up. Among those with HbA1c ≥7% (≥53 mmol/mol) during 1 year post diagnosis, only 40% received IT before 12 months, and the median time to IT was 16 months (Table 2). In patients with HbA1c ≥7% (≥53 mmol/mol) consistently during 2 years post diagnosis, only 64% patients received IT before 2 years of diagnosis, and the median time to IT or receiving at least two OADs was 17 months (mean = 23 months).

**Table 2:** Proportions of patients with HbA1c above 7 and 7.5% consistently during 1 year and 2 years post diagnosis of diabetes by categories of time to intensified treatment, and the median (IQR) months to treatment intensifications from diagnosis of diabetes for various classifications of Hba1c trajectory

	n (%) by time to intensified treatment categories			Time to intensification median (IQR), months		
	<6 months	<1 year	<2 years	Time to IT	Time to 2 OADs	Time to 3 ADDs
HbA1c ≥7% (≥53 mmol/mol)						
Consistently during 1 year	5,768 (27)	8,776 (40)	14,101 (65)	16 (5, 32)	17 (6, 32)	37 (20, 60)
Consistently during 2 years	4,696 (28)	6,864 (40)	10,904 (64)	17 (5, 31)	17 (6, 32)	36 (20, 58)
HbA1c ≥7.5% (≥58 mmol/mol)						
Consistently during 1 year	3,783 (29)	5,869 (46)	9,284 (72)	14 (5, 26)	14 (5, 27)	33 (18, 54)
Consistently during 2 years	2,774 (32)	3,999 (46)	6,171 (70)	14 (4, 27)	15 (5, 28)	31 (18, 52)

Among patients with HbA1c ≥7.5% (≥58 mmol/mol) consistently during 1/2 year post diagnosis, 23/22% never received any IT during follow-up. Among those with HbA1c ≥7.5% (≥58 mmol/mol) during 1 year post diagnosis, only 46% received IT before 1 year, and the median time to IT or receiving at least two OADs was 14 months (mean 20 months). In patients with HbA1c ≥7.5% (≥58 mmol/mol) consistently during 2 years, 70% received IT within 2 years post diagnosis. The median/mean months to IT and 3 ADDs in this group were 14/20 and 31/38 months, respectively.

The distribution of patients with diagnosis of diabetes over different time periods from 1990 to 2012 are presented in Table 1. The average HbA1c at diagnosis in patients who were diagnosed before 2005, between 2005 and 2009 and from January 2010 onwards were 8.37, 8.02 and 7.81%, respectively, with similar standard deviation of 2.2. Among patients with HbA1c above 7.5% (58 mmol/mol) consistently over 1 year post diagnosis of diabetes, adjusting for age and baseline HbA1c, patients diagnosed between 2005 and 2009 and from January 2010 onwards were 46% and 139% more likely to receive intensified treatment, compared to those who were diagnosed prior to January 2000.

Among patients with HbA1c above 7.5% (58 mmol/mol) consistently for 1 year from diagnosis of diabetes—patients older than 70 years at diagnosis, with renal disease and with cardiovascular disease were 30% (95% CI of odds ratio: 0.63, 0.76), 13% (95% CI of odds ratio: 0.79, 0.96) and 50% (95% CI of odds ratio: 0.45, 0.57) less likely to receive intensified treatment during follow-up, respectively.

### Effect of clinical inertia on cardiovascular risk

During 5.3 years of median follow-up, the proportions of patients who experienced at least one episode of MI, stroke, HF, and any of composite CVE were 2.3, 3.0, 2.2 and 6.8%, respectively. Among those with a history of CVD (n = 11,955), these proportions were 4.8, 7.5, 4.1 and 14.4%, respectively. Patients with HbA1c above 7% (53 mmol/mol) consistently during 1 year post diagnosis had significantly higher rate (per 1,000 person years) of composite CVE [rate (95% CI) for HbA1c ≥7% (≥53 mmol/mol) vs <7% (<53 mmol/mol): 1.15 (1.10, 1.20) vs 1.04 (1.01, 1.08)]. The event rates were similar for those with HbA1c ≥7.5% (≥58 mmol/mol) during 1 year post diagnosis.

Among all patients, compared to patients with HbA1c below 7% (53 mmol/mol) who received IT before 1 year of diagnosis, patients with HbA1c ≥7% (≥53 mmol/mol) not receiving IT within a year had significantly increased risk of MI, HF, stroke and composite CVE significantly by 67, 64, 51 and 62%, respectively, after adjusting for various confounding factors (all p < 0.01, Table 3). Among patients without history of any CVD (n = 93,522), a delay in treatment intensification by 12 months [in conjunction with poor HbA1c level above 7% (53 mmol/mol)] was associated with significantly increased risks for MI, HF, stroke and composite CVE by 80% (HR CI: 1.45, 2.22), 63% (HR CI: 1.36, 1.96), 50% (HR CI: 1.22, 1.84) and 64% (HR CI: 1.45, 1.85), respectively (all p < 0.01). Delay in treatment intensification by 12 months in interaction with poor HbA1c level above 7.5% (58 mmol/mol) during 1 year post diagnosis also had similar increased risks for CVE. Among patients with history of CVD prior to diagnosis of diabetes (n = 11,955), delay in treatment intensification in conjunction with poor glycaemic control was also significantly associated with increased risk of HF and composite CVE, but not with MI or stroke (Table 3).

**Table 3:** Hazard ratios (95% CI) associated with delays in treatment intensification by 1 year in interaction with poor glycaemic control [HbA1C ≥7% (≥53 mmol/mol) and HbA1C ≥7.5% (≥58 mmol/mol)] consistently during 1 year post diagnosis of diabetes for cardiovascular events

	Without previous CVD HR (95% CI)	P	With previous CVD HR (95% CI)	P	All patients HR (95% CI)	P
MI						
With HbA1C ≥7% (≥53 mmol/mol)						
A: IT within 1 year	1.37 (1.09, 1.72)	<0.01	1.21 (0.81, 1.82)	0.36	1.32 (1.08, 1.61)	<0.01
B: IT after 1 year	1.80 (1.45, 2.22)	<0.01	1.34 (0.91, 1.96)	0.13	1.67 (1.39, 2.01)	<0.01
With HbA1C ≥7.5% (≥58 mmol/mol)						
A: IT within 1 year	1.59 (1.27, 1.99)	<0.01	1.12 (0.73, 1.71)	0.62	1.47 (1.20, 1.79)	<0.01
B: IT after 1 year	1.56 (1.24, 1.97)	<0.01	1.42 (0.95, 2.14)	0.09	1.52 (1.24, 1.86)	<0.01
HF						
With HbA1C ≥7% (≥53 mmol/mol)						
A: IT within 1 year	1.14 (0.94, 1.40)	0.19	1.52 (1.09, 2.12)	0.015	1.23 (1.04, 1.46)	0.017
B: IT after 1 year	1.63 (1.36, 1.96)	<0.01	1.66 (1.21, 2.27)	<0.01	1.64 (1.40, 1.91)	<0.01
With HbA1C ≥7.5% (≥58 mmol/mol)						
A: IT within 1 year	1.32 (1.07, 1.62)	<0.01	1.52 (1.08, 2.13)	0.016	1.37 (1.15, 1.63)	<0.01
B: IT after 1 year	1.61 (1.32, 1.97)	<0.01	1.50 (1.06, 2.12)	0.021	1.58 (1.33, 1.88)	<0.01
Stroke						
With HbA1C ≥7% (≥53 mmol/mol)						
A: IT within 1 year	1.28 (1.03, 1.60)	0.025	1.80 (1.10, 2.95)	0.019	1.36 (1.12, 1.66)	<0.01
B: IT after 1 year	1.50 (1.22, 1.84)	<0.01	1.60 (0.98, 2.60)	0.06	1.51 (1.25, 1.83)	<0.01
With HbA1C ≥7.5% (≥58 mmol/mol)						
A: IT within 1 year	1.27 (1.01, 1.59)	0.040	1.89 (1.16, 3.06)	0.010	1.36 (1.11, 1.67)	<0.01
B: IT after 1 year	1.37 (1.09, 1.71)	<0.01	1.34 (0.78, 2.30)	0.28	1.36 (1.11, 1.67)	<0.01
Any CVE						
With HbA1C ≥7% (≥53 mmol/mol)						
A: IT within 1 year	1.22 (1.07, 1.38)	<0.01	1.36 (1.06, 1.74)	0.015	1.24 (1.11, 1.40)	<0.01
B: IT after 1 year	1.64 (1.45, 1.85)	<0.01	1.57 (1.25, 1.98)	<0.01	1.62 (1.46, 1.80)	<0.01
With HbA1C ≥7.5% (≥58 mmol/mol)						
A: IT within 1 year	1.32 (1.15, 1.50)	<0.01	1.40 (1.09, 1.81)	<0.01	1.33 (1.19, 1.50)	<0.01
B: IT after 1 year	1.50 (1.32, 1.71)	<0.01	1.50 (1.17, 1.94)	<0.01	1.50 (1.33, 1.68)	<0.01

The reference group was those with TTIT <12 months and HbA1c <7 or 7.5% (<53 or 58 mmol/mol). A: TTIT <12 months and HbA1c ≥7 or 7.5% (≥53 or 58 mmol/mol), B: TTIT ≥12 months and HbA1c ≥7 or 7.5% (≥53 or 58 mmol/mol). Analyses are based on multivariate Cox-regression models, with contrast matrix to evaluate the effect of delay in treatment intensification in conjunction higher and lower levels of HbA1c at 7 and 7.5% cut offs (53 and 58 mmol/mol).

Among patients with HbA1c above 7% (53 mmol/mol) consistently during 2 years post diagnosis (n = 23,101), patients who did not receive intensified treatment had 82% (HR CI: 1.67, 2.10) increased risk of composite CVE. The risk estimates were similar for patients with and without history of cardiovascular diseases. These estimates were obtained after adjusting for all factors mentioned in the method section, except any adjustment for HbA1c levels.

Irrespective of glycaemic control, failure to intensify anti-hyperglycaemic treatments was associated with 42% (HR CI: 1.21, 1.66) and 48% (HR CI: 1.36, 1.61) significantly increased risk of CVE among patients with and without the history of cardiovascular diseases, respectively.

Subgroup analyses with adjustments for available data on BMI, systolic blood pressure, LDL-cholesterol and total cholesterol at diagnosis revealed similar risks on cardiovascular outcomes, associated with clinical inertia.

### Additional analyses on the effects of glycaemic burden

Among patients receiving IT before 12 months, those with HbA1c above 7% (53 mmol/mol) and 7.5% (58 mmol/mol) during 1 year post diagnosis had significantly increased risk of any CVE by 24% (HR CI: 1.11, 1.40) and 33% (HR CI: 1.19, 1.50), respectively compared to those with HbA1c below 7% (53 mmol/mol) and 7.5% (58 mmol/mol) (Table 3). These patients also had significantly increased risks for MI, HF and stroke. Irrespective of the intensity of anti-diabetes drugs and the history of CVD, patients with HbA1c above 7% (53 mmol/mol) consistently over 1 year post diagnosis had 21% (HR CI: 1.15, 1.28; p < 0.01) increased risk of composite CVE. Among patients without history of CVD, HbA1c above 7% (53 mmol/mol) was associated with 22% increased risk for CVE (HR CI: 1.14, 1.29; p < 0.01).

Male patients, current smokers, and patients who developed renal disease during follow-up had 12, 51, and 43% increased risk of composite CVE (all p < 0.01), respectively.

## Discussion

Our population level study, based on more than 100,000 newly diagnosed T2DM patients with median 5.3 years of follow-up, reveals that (1) 26% of patients with HbA1c above 7% (53 mmol/mol) during 2 years post diagnosis of diabetes did not receive any intensified treatment for hyperglycaemia during follow-up, (2) 32 and 46% of patients receiving early treatment intensification within 6 and 12 months of diagnosis continued to have poor glycaemic control over 2 years post diagnosis [HbA1c above 7.5% (58 mmol/mol)], (3) a 1 year delay in treatment intensification in conjunction with poor glycaemic control significantly increased the risks of HF, stroke and composite CVE in patients with and without history of CVD before diagnosis of diabetes, and (4) irrespective of early treatment intensification, the glycaemic burden was significantly associated with increased risk of MI, HF, stroke and composite CVE.

In the study cohort 54% never received any intensified treatment, while their average HbA1c level remained above 6.5% (48 mmol/mol), but below 7% (53 mmol/mol), during 2 years post diagnosis (Fig. 1a). In the “never intensified” group, the proportions of patients with HbA1c consistently above 7% (53 mmol/mol) and 7.5% (58 mmol/mol) during 1/2 years post diagnosis were 15/11% and 7/4%, respectively. The median follow-up time in this group was 4.7 years. The incidence of any CVE in this group was 5.5% compared to 8.4% in the IT group.

Earlier observational studies have reported about 3 years of delay in treatment intensification (irrespective of glucose level) [21], high glycaemic burden [HbA1c >7.5% (>58 mmol/mol)] for over more than 5 years after the addition of a multiple OADs and insulin [19, 21, 34, 35], and that it took several years to add insulin after the initiation of 2 OADs [34]. The median (IQR) months to treatment intensification in our study was 21 [5, 44], which is about 12 months less than that reported by Khunti et al. [21]. However, our results are not directly comparable, as the studies were differently designed. In our study those who received IT within 6/12 months of diagnosis had a significant reduction in HbA1c by 2.5/2.3% within 6 and 12 months post diagnosis (Fig. 1b). Our findings on the failure of intensified treatment to maintain HbA1c within a clinically acceptable limit is broadly in line with those reported in the large surveys conducted in USA and UK [36–39]. However, as no study has yet reported the trajectory of HbA1c with treatment intensification, our unique findings are not directly comparable. The mechanisms of increased risk of macrovascular events with hyperglycaemia are not fully known but may include oxidative stress, inflammation and thrombosis [40–42].

The ACCORD study in patients with 10 years of duration of T2DM could not establish any significant benefit of intensified glucose lowering treatment on macrovascular event during 3.5 years of median follow-up [6]. However, the meta analyses of four major cardiovascular outcome trials in patients with T2DM, including the ACCORD trial data, reported a 15–17% risk reduction in MI, 15% risk reduction in coronary heart disease and 9% risk reduction in major cardiovascular event associated with intensive glucose control therapy [9, 43]. While the UKPDS trial was based on newly diagnosed diabetes patients, the median duration of diabetes in the participants of ACCORD, ADVANCE and VADT trials was 9 years, and 34% of them had history of major cardiovascular disease before randomisation. Findings of our study based on a cohort of patients with new diagnosis of T2DM is broadly in line with the findings of the meta analyses in relation to the beneficial effects of intensified glucose lowering treatments on major cardiovascular risks. The recent cardiovascular outcome trials in established T2DM patients had about 5 years of follow-up and do not provide insight into any longer time benefit in terms of cardiovascular risk. In our cohort with minimum 5 years of follow-up (n = 47,161) who continued to have HbA1c above 7% over 1 year, delay in treatment intensification was associated with 65% (95% CI of HR: 1.49, 1.84) increased risk of CVE—very similar to what we observed in the whole cohort with median 5.3 years of follow-up.

Although early treatment intensification did not reduce the HbA1c below clinically acceptable limit in a significant proportion of patients, the residual benefit of intensified treatment on macrovascular risk is considerable. Compared to patients with HbA1c <7% (<53 mmol/mol) during 1 year post diagnosis, those with HbA1c ≥7% (≥53 mmol/mol) and received IT within a year of diagnosis had 38% lower risk for any CVE (HR: 1.24) compared to those who had delayed treatment intensification (HR: 1.62, Table 3). Early treatment intensification also showed considerable residual benefits on MI, HF and stroke. This new finding on the residual benefit of intensive anti-diabetes treatment on long-term macrovascular risk, even in patients with continued high glycaemic burden, needs further evaluations with longer term HbA1c trajectory, and other cardiovascular risk factors.

Treatment intensification by insulin and sulfonylurea drugs and its association with cardiovascular and mortality risk was recently evaluated by Roumie et al. [44]. Patients who added insulin to the first line metformin treatment had 30% significantly higher adjusted risk of composite macrovascular events, compared to those who intensified the treatment by adding sulfonylurea to metformin. In our study about 9% patients received treatment intensification with insulin, and these patients had higher HbA1c at diagnosis and during follow-up before intensification, as expected. Patients receiving insulin treatment are generally at higher risk profile, and the finding from Roumie et al. [44] could be partially attributed to this factor. In this study our focus was on glycaemic burden and the failure to intensify anti-hyperglycaemic treatment when deemed necessary. We do however recognise the importance of evaluating therapeutic inertia in a holistic way including cardiovascular risk burden. In our risk analyses, we have adjusted for the use of cardio-protective medications (including anti-hypertensive and lipid lowering drugs) and the risk of renal diseases. We also conducted separate analyses adjusting for available cardiovascular risk factors (weight, blood pressure and cholesterol) in 62,000 patients.

While MI and stroke are relatively well defined hard outcomes with acute onset, HF is a disease of slow onset which develops over time. This introduces the difficulty in defining a correct time of onset, and the likelihood of getting HF detected in the setting of primary care database to a great extent depends on medical attention and on the number of encounters with general practitioners. To account for possible detection bias in this context, we conducted additional analyses with HF confirmed by electrocardiogram analyses, resulting in similar risk estimates for both events.

Patient-level data from electronic databases present challenges in terms of accuracy and completeness of the study variables of interest. The limitations of this study include: (1) the time to treatment intensification was based on the dates of available prescriptions longitudinally, with the likelihood of missing data; (2) non-availability of adherence data on medications; (3) non-availability of prescription on diet and exercise; (4) failure to account for the socio-economic status of the patients, which may well be associated with poor glycaemic control and with an elevated risk of cardiovascular diseases; (5) non-availability of complete and reliable data on alcohol consumption; (6) non-availability of complete and reliable longitudinal data on doses for individual medications, (7) non-availability of hospitalisation data for MI and stroke, and (8) the potential for residual confounding, as common in any clinical epidemiological study. Due to lack of data on longitudinal doses of individual anti-diabetes medications, we could not consider the dose escalation for some anti-diabetes therapies as potential treatment intensification. However, these issues are unlikely to affect the robustness of the results of this study. The large analysis cohort was selected from the validated CPRD database should be considered as a representative sample, and as such, provides a good picture of the state of diabetes control in routine practice. Apart from complete data on demographics, we had complete data on HbA1c measured within 3-month window of diagnosis of diabetes. The 6-monthly follow-up measures of HbA1c were imputed for only 9% missing cases with random missing pattern. Finally, careful design of the study by defining sensible exposure time, time line for events, and appropriate adjustments for various aspects while determining the time-to-events are the primary strengths of the study.

## Conclusions

As with all observational studies, we were unable to provide definitive evidence for direct cause–effect relationships, and the risks of false positive or negative findings due to selection bias and residual confounding, even after the most stringent corrections, may not be trivial. However, although the results from large controlled clinical trials evaluating the effect of intensified glucose lowering treatment on macrovascular complications in patients with T2DM have been inconclusive, our longitudinal study with primary care level data demonstrates the beneficial effect of guideline recommended tight glycaemic targets on long-term cardiovascular risks.

## Abbreviations

ADD: anti-diabetes drug
BMI: Body mass index
CI: confidence interval
CPRD: Clinical Practice Research Database
CVE: macrovascular event
HbA1c: haemoglobin A1c
HF: heart failure
HR: hazard ratio
INS: insulin
IT: treatment intensification
MI: myocardial infarction
OAD: oral antidiabetes drug
OR: odds ratio
T2DM: type 2 diabetes
TTIT: time to treatment intensification
